# Intrinsic functional brain connectivity changes following aerobic exercise, computerized cognitive training, and their combination in physically inactive healthy late-middle-aged adults: the Projecte Moviment

**DOI:** 10.1007/s11357-023-00946-8

**Published:** 2023-10-23

**Authors:** Stavros I. Dimitriadis, Alba Castells-Sánchez, Francesca Roig-Coll, Rosalía Dacosta-Aguayo, Noemí Lamonja-Vicente, Pere Torán-Monserrat, Alberto García-Molina, Gemma Monte-Rubio, Chelsea Stillman, Alexandre Perera-Lluna, Maria Mataró

**Affiliations:** 1 Department of Clinical Psychology and Psychobiology, University of Barcelona, Passeig Vall d’Hebron 171, 08035 Barcelona, Spain; 2 Institut de Neurociències, University of Barcelona, Barcelona, Spain; 3 Unitat de Suport a La Recerca Metropolitana Nord, Fundació Institut Universitari Per a La Recerca a L’Atenció Primària de Salut Jordi Gol I Gurina, Mataró, Spain; 4 Institut d’Investigació en Ciències de La Salut Germans Trias I Pujol (IGTP), Badalona, Spain; 5 Institut de Recerca Sant Joan de Déu, Esplugues de Llobregat, Spain; 6 Department of Medicine, Universitat de Girona, Girona, Spain; 7 Institut Guttmann, Institut Universitari de Neurorehabilitació, Universitat Autònoma de Barcelona, Badalona, Spain; 8 Centre for Comparative Medicine and Bioimage (CMCiB), Germans Trias I Pujol Research Institute (IGTP), Badalona, Spain; 9 Department of Psychology, University of Pittsburgh, Pittsburgh, PA USA; 10 B2SLab, Departament d’Enginyeria de Sistemes, CIBER-BBN, Automàtica I Informàtica Industrial, Universitat Politècnica de Catalunya, 08028 Barcelona, Spain; 11 Department of Biomedical Engineering, Institut de Recerca Pediàtrica Hospital Sant Joan de Déu, 08950 Esplugues de Llobregat, Barcelona, Spain

**Keywords:** Aerobic exercise, Computerized cognitive training, Combined training, fMRI, Resting-state functional connectivity, Default mode network, Multiplexity

## Abstract

Lifestyle interventions have positive neuroprotective effects in aging. However, there are still open questions about how changes in resting-state functional connectivity (rsFC) contribute to cognitive improvements. The Projecte Moviment is a 12-week randomized controlled trial of a multimodal data acquisition protocol that investigated the effects of aerobic exercise (AE), computerized cognitive training (CCT), and their combination (COMB). An initial list of 109 participants was recruited from which a total of 82 participants (62% female; age = 58.38 ± 5.47) finished the intervention with a level of adherence > 80%. Only in the COMB group, we revealed an extended network of 33 connections that involved an increased and decreased rsFC within and between the aDMN/pDMN and a reduced rsFC between the bilateral supplementary motor areas and the right thalamus. No global and especially local rsFC changes due to any intervention mediated the cognitive benefits detected in the AE and COMB groups. Projecte Moviment provides evidence of the clinical relevance of lifestyle interventions and the potential benefits when combining them.

**Supplementary Information:**

The online version contains supplementary material available at 10.1007/s11357-023-00946-8.

## Introduction

“Might strength, efficiency, and segregation update, if you challenge yourself.” Late adulthood is known for being the finest moment for crystallized cognition; older adults maintain or even increase vocabulary and expertise-based abilities. However, even healthy normal aging, characterized by the lack of pathology, is related to a progressive decline in fluid cognitive abilities, which is associated with reductions in executive function, memory, attention, and processing speed [22, 40, 41, 67, 75, 76, 91, 113, 126–128, 172] and deficits in social and independent functioning [77]. Concurrently, one of the well-documented hallmarks of aging is the alteration of brain health. Extensive literature focused on the structural changes, such as loss of gray matter volume and less white matter integrity, and their association with age-related cognitive changes [61, 120, 177] and observed decline in brain function.

Functional brain connectivity emerged as a new promising approach based on the fact that cognitive functions are the result of coordinated activity among distant brain areas that work in networks [124]. Functional magnetic resonance imaging (fMRI) allows for examining temporal synchrony derived from a time-series covariance of blood-oxygen-level dependent (BOLD) signals from multiple regions of interest (ROIs) [13, 162]. In particular, resting-state fMRI (rs-fMRI) aims to identify coherences in the spontaneous fluctuations of low-frequency oscillations of the BOLD signal at rest [19] which might be informative of the brain architecture and individual differences [65]. Studies found that the brain is organized into a number of networks that show high within-network connectivity and lesser long-range connections between networks in order to maximize its efficiency [12, 24, 102]. Although several networks have been characterized across subjects using rs-fMRI [43] for several aspects [77], the seven most relevant resting-state networks (RSNs) were defined by Yeo et al. [178]: the default mode network (DMN), the frontoparietal control network (FPCN), the cingulo-opercular network (CON) or salience network (SN), the dorsal attention network (DAN), the limbic network (LN), the visual network (VN), and the sensorimotor network (SMN) [77, 103, 124, 162]. A general finding is reduced functional connectivity in older adults compared to younger ones [124] which reflects the reorganization of the brain networks during aging [79]. Aging is related to a neural reorganization process characterized by bilateral hemispheric activation of the solicited regions (hemispheric asymmetry reduction in older adults model) and decreased functional selectivity and more diffuse and less specialized functional connectivity (compensation-related utilization of neural circuits hypothesis model) [54, 66, 69, 83, 85, 102, 118] which has been found to be steeper in clinical populations [49, 62]. All RSNs experience a certain degree of deterioration in normal aging, but especially the higher-order cognitive networks [124]. Studies found decreased within-network connectivity on the DMN, CON, sensorimotor network (SMN), fronto-parietal network (FPN) and dorsal attention network (DAN), increased connectivity in between networks, less segregated network structure and local efficiency, and higher participation coefficient using different types of connectivity analyses [2, 5, 17, 31, 42, 45, 66, 72, 80, 82, 87, 102, 112, 124, 138, 155, 162, 168]. Ongoing research has commonly associated the higher-order cognitive networks (DMN, FPN, and CON) with deficits in executive function, attention, processing speed, and memory [2, 31, 42, 66, 87, 112, 124, 133, 168]. Changes are not that consistent for the somatosensory, motor, and subcortical networks which might remain relatively stable in older adults [124]. Besides, studies involving middle-aged adults found that a decline in connectivity becomes progressively evident [136] with a decrease in segregation [53]. This fact suggests that middle age might be a critical moment to prevent or delay the inversion of the functional connectivity trajectories and, in turn, promote cognitive health. Results suggest that the last developed areas are the first to be altered, coherent with the hypothesis “last in, last out” [117]. Nevertheless, a more integrative approach, the Scaffolding Theory of Aging and Cognition (STAC-r), proposed that multiple factors might influence the age-related decline and an active lifestyle contributes to the scaffolding of novel compensatory networks. This model included behavioral interventions, such as physical and cognitive training, as potential mechanisms to preserve cognitive and brain health and, fortunately, emerging evidence indicates that age-related decline may not be inevitable [143].

Different types of exercise have been related to cognitive and brain health. In particular, evidence highlights the role of moderate-intensity aerobic exercise (AE) in the promotion of executive function, processing speed, attention, and memory in older adults [9, 38, 58, 110]. The mechanistic hypotheses of the observed cognitive benefits are multiple and were organized in a three-level model (molecular–brain–behavioral) by Stillman (2019) including clear influences of individual variables such as sex and age. At the brain level, while some papers have focused on structural brain changes reporting promising benefits on the volume-specific regions such as the prefrontal cortex and the hippocampus after mid-term interventions [30, 56], others have focused on AE-related changes in the functional brain connectivity [173]. First evidence suggesting an association between physical activity and cardiorespiratory fitness (CRF) with increased coherence in large-scale RSNs, such as DMN, was obtained in cross-sectional samples [163, 165, 167]. Researchers translated this experience to randomized controlled trials (RCT) in order to better understand inconsistencies across different protocols and explore how AE applied as a controlled intervention might produce similar results [143]. Although existing RCTs are highly variable and scarce, recent systematic reviews concluded that AE is a viable strategy for modifying functional brain connectivity and perfusion of the hippocampus [34, 57, 143, 150]. Up to now, the most consistent results are found in studies including mid and long-term AE interventions. The longest study, lasting 12 months, found increased efficiency in the DMN, FP, and frontal-executive (FE) networks [166]. Six months of AE intervention resulted in increased connectivity between the dorsolateral prefrontal cortex and the superior parietal lobe [116] and a significant association between increased CRF and functional connectivity (FC) between the brain areas of the DMN, although no significant changes were found after the AE in a cross-sectional study [145]. Significant results have also been reported after 4 months of interventions showing increased efficiency in the parahippocampus [157], cerebral blood flow in the hippocampus [84], and the connectivity between the hippocampus and anterior cingulate cortex [149]. Shorter AE interventions have shown more inconsistent results. For example, the study of Maass et al. [101] found increased perfusion in the hippocampus while Chapman et al. [32] reported non-significant changes in this area but significant in the bilateral anterior cingulate cortex. Three months of cycling have been related to increased rsFC between the DMN and motor regions [106], but 3 months of walking did not lead to significant changes in connectivity between the precuneus and frontal-parietal cortices [35]. These results suggest that shorter interventions might be enough to observe changes in brain function, although parameters of the physical activity might be better specified. Since all previous 3-month RCTs are scheduled mostly 3 days, we consider that studying changes in brain function in a short-term but following a high-frequency (5 days) AE intervention might shed light on these inconsistencies.

Cognitive training is another example of the most studied behavioral intervention. Computerized cognitive training (CCT), which refers to single cognitive training tasks performed on electronic devices, became a promising approach to promote cognition and structural and functional brain health [151, 154]. Evidence related CCT to benefits in global cognition as well as specific trained functions such as verbal memory, [6, 8, 131], processing speed [92], and executive function [8]. These results highlighted the potential neuroplasticity even of the aging brain to strengthen or maintain synaptic connections and brain function. Functional brain imaging became a useful technique to describe the neuroplastic mechanisms underlying CCT cognitive effects [15]. Most of the papers used a task-fMRI approach, which generally identified decreased activity in the areas functionally involved in the trained task interpreted as more neural efficiency [47, 81, 86]. However, literature regarding changes in the spontaneous fluctuations after CCT interventions specifically in healthy older adults is scarce [26, 96, 99]. For a detailed review, see van Balkom et al. [159]. Results from previous RCTs found patterns of increased or decreased rsFC involving areas of the DMN, CEN, and DAN after CCT [33, 93, 122, 146]. Only one research project including 3 months of multimodal cognitive training applied in the hospital by experts studied the effects of the intervention in multiple age-sensitive networks [26]. Results showed a maintained or increased anterior–posterior and interhemispheric rsFC within the DMN, CEN, and SN, a maintained DMN-SN coupling, an anti-correlation pattern between DMN and CEN [26] and a more integrated local FC in the training group than controls [48]. However, to our knowledge, the extent of these effects on home-based computerized multimodal training has not been studied before. We consider that addressing this gap could support CCT as a promising intervention able to counteract age-related rs-FC decline.

AE and CT showed promising results and a certain degree of complementarity in matters of mechanisms involved in the observed cognitive benefits [153]. Exercise impacts most of the body systems promoting low inflammation and oxidative stress, cardiovascular adaptations, and neural repairing responses that might be enhanced by the stimulation and regulation of neuroplasticity through cognitive stimulation [59, 111]. Although evidence is still too few and sometimes inconsistent, systematic reviews argue in favor of an advantage when combining AE and CT [7, 83, 90]. Results show that general cognitive function [132], executive function [55], processing speed, and memory [60] benefit from a combined intervention (COMB) and changes in brain structure [97] and function have been reported.

Six months of combined training have been shown to produce changes in the strength of functional connectivity of the precuneus, right angular gyrus, and posterior cingulate cortex in the DMN and the left frontal eye field in the DAN [115]. Moreover, six months of COMB specifically impacts the connectivity between the medial prefrontal cortex and medial temporal lobe in the DMN [95]. Up to now, just one published paper explored changes in brain function after a shorter period of time but used a task-related fMRI approach. A dual-task training involving AE and working memory training for 12 weeks reported increased brain activity around the bilateral temporoparietal junctions which are highly related to attentional processes, while participants were performing a working memory task in the scanner [148]. Based on this positive evidence, and the lack of currently published results showing changes in rs-fMRI after short-term COMB interventions, we consider it imperative to analyze potential brain function changes at rest.

Projecte Moviment is an RCT protocol that involved the effect of a high-frequency (5 days per week) short-term (12 weeks) program of AE, computerized cognitive training (CCT), and their combination in healthy physically inactive older adults [29]. The observed changes in cognition, psychological status, physical activity, molecular biomarkers, and brain volume outcomes have been published in Roig-Coll et al. [121] and Castells-Sánchez et al. [28].

In this study, (1) we aim to assess changes in functional connectivity on rs-fMRI related to intervention, (2) we intend to investigate the moderating role of sex and age on functional connectivity on rs-fMRI changes, and (3) the possibility that changes in functional connectivity on rs-fMRI outcomes mediate the relationship between the intervention and cognitive benefits.

## Methods

### Study design

Projecte Moviment is a multi-center, single-blind, proof-of-concept RCT recruiting healthy low active late-middle-aged adults to be assigned in a four parallel-group design including 12-week intervention programs. Participants were assessed at baseline and at trial completion and randomly assigned to an AE group, a CCT group, a COMB group, and a waitlist control group. The study was developed by the University of Barcelona in collaboration with Institut Universitari d’Investigació en Atenció Primària Jordi Gol, Hospital Germans Trias i Pujol and Institut Guttmann and approved by the responsible ethics committees (Bioethics Commission of the University of Barcelona — IRB00003099 — and Clinical Research Ethics Committee of IDIAP Jordi Gol — P16/181) following the Declaration of Helsinki. The study took place between November 2015 and April 2018.

This research paper follows the previously registered (ClinicalTrials.gov; NCT031123900) and published protocol [29]. Results on the primary and partial secondary hypotheses [27, 121] would be included for discussion.

### Participants

Healthy adults aged 50 to 70 years old from the Barcelona metropolitan area were recruited using multiple strategies (lists of patients of general physicians, volunteers from previous studies, oral presentations in community centers, advertisements, and local media). Volunteers were informed and screened over the phone and in an on-site interview, and those meeting inclusion and exclusion criteria (see Table 1) signed a written informed consent prior to the study involvement.

**Table 1:** Inclusion and exclusion criteria for Projecte Moviment

Inclusion criteria	Exclusion criteria
Aged 50–70 years	Current participation in any cognitive training activity or during last 6 months > 2 h/week
≤ 120 min/week of physical activity during last 6 months	Diagnostic of dementia or mild cognitive impairment
Mini-Mental State Examination (MMSE) ≥ 24	Diagnostic of neurological disorder: stroke, epilepsy, multiple sclerosis, traumatic brain injury, brain tumor
Montreal Cognitive Assessment 5-min (MoCA 5-min) ≥ 6	Diagnostic of psychiatric illness current or during last 5 years
Competency in Catalan or Spanish	Geriatric Depression Scale (GDS-15) > 9
Adequate visual, auditory, and fine motor skills	Consumption of psychopharmacological drugs current or during last 5 years; or more than 5 years throughout life
Acceptance of participation in the study and signature of the informed consent	History of drug abuse or alcoholism current or during last 5 years; or more than 5 years throughout life; > 28 men and > 18 woman unit of alcohol/week
	History of chemotherapy
	Contraindication to magnetic resonance imaging

We randomly assigned participants to AE, CCT, COMB, and waitlist control groups after baseline assessments using a random combination of selected demographic variables (sex, age, and years of education) in order to obtain balanced groups. The allocation sequence was designed by a statistician and the intervention team was responsible for the allocation. Professionals involved in the assessment remained blind to group assignment.

### Interventions

Participants assigned to intervention groups went through home-based programs lasting 12 weeks, 5 days per week. Participants randomized to the waitlist control group were on the waitlist for 12 weeks and were asked not to alter their regular lifestyle.

The AE intervention program consisted of a progressive brisk walking program (Week 1: 30 min per day at 9–10 on the Borg Rating of Perceived Exertion Scale (BRPES; [20] perceived as light intensity. Week 2: 45 min per day at 9–10 on BRPES. Week 3 to 12 (10 weeks): 45 min per day at 12–14 on BRPES perceived as moderate-high effort). The CCT intervention program consisted of multimodal cognitive training scheduled in sessions of 45 min. Participants used Guttmann Neuropersonal Trainer online platform (GNPT®, Spain; Solana et al., 2014, 2015) and performed tasks involving executive function, visual and verbal memory, and sustained, divided, and selective attention. Baseline cognitive performance and the ongoing scores of the activities were used by the GNPT platform to adjust the demand of the activities. The COMB intervention program consisted of a combination of the brisk walking program and the CCT as described above, separately, in single continuous bouts of 45 min for each intervention 5 days per week without order or time-point restrictions.

The intervention team was available for participants, registered participants’ activity (phone calls every 2 weeks and a midpoint visit, and a final visit) and ensured participation in solving inconveniences and barriers, and obtained adherence based on participants’ feedback and platform data. Participants monitored their activity in a diary, registering the date and duration of the activity and any adverse events occurring as well as the intensity of the walking in BRPES units.

The protocol for each intervention condition is explained in more detail elsewhere [29].

### Assessment

Participants went through all assessments in clinical environments at baseline within 2 weeks prior to the start of the intervention and, again, within 2 weeks after the completion of the program [29].

#### Neuroimaging: resting-state functional MRI data acquisition

##### The Projecte Moviment

MRI data were collected using a 3 T Siemens Magnetom Verio Symo MR B17 (Siemens 243 Healthineers, Erlangen, Germany) located at the Hospital Germans Trias i Pujol. Participants were asked to rest with their eyes closed during the scan session, had head motion constrained, and were offered earplugs to reduce the adverse effects of scanner noise. Resting-state functional BOLD imaging scans were obtained with a gradient echo planar imaging sequence (acquisition time 8:08 min, voxel 3.1 × 3.1 × 3.0 mm, TR/TE 2000/25 ms, flip angle 78°, slices 39, thickness 3 mm, volumes 240). We also collected T1-weighted multi-planar reformat sequences (acquisition time 5:26 min, voxel 0.9 × 0.9 × 0.9 mm, TR/TE/TI 1900/2.73/900 ms, flip angle 9°, slices 192, thickness 0.9 mm) which were used during the rs-fMRI preprocessing to co-register functional and structural MRI data. Scans were visually checked by an expert neuroradiologist.

##### The NYU dataset

For evaluating the stability of our findings based on the adopted preprocessing pipeline and functional network construction, we analyzed an open test–retest rs-fMRI dataset. This is an open dataset from the International Neuroimaging Data-Sharing Initiative (INDI) (http://www.nitrc.org/projects/nyu_trt) that was originally described in [134]. The NYU dataset includes 25 participants (mean age 30.7 ± 8.8 years, 16 females) with no history of psychiatric or neurological illness. Three resting-state scans were acquired from each participant. Scans 2 and 3 were conducted in a single session with 45 min apart, while the scan 1 took place on average 11 months (range 5–16 months) after scans 2 and 3.

Each scan was acquired using a 3 T Siemens (Allegra) scanner and consisted of 197 contiguous EPI functional volumes (TR = 2000 ms; TE = 25 ms; flip angle = 90°; 39 axial slices; field of view (FOV) = 192 × 192 mm^2^; matrix = 64 × 64; acquisition voxel size = 3 × 3 × 3 mm^3^). Participants were instructed to remain still with their eyes open during the scan. For spatial normalization and localization, a high-resolution T1-weighted magnetization prepared gradient echo sequence was also obtained (MPRAGE, TR = 2500 ms; TE = 4.35 ms; TI = 900 ms; flip angle = 8°; 176 slices, FOV = 256 mm).

#### Resting-state functional MRI preprocessing, and denoising

The preprocessing of the rs-fMRI data from both datasets, “Projecte Moviment” and NYU, was conducted following the same standard workflow implemented in the CONN toolbox (http://www.nitrc.org/projects/conn), version 17f [174]. A standard pipeline of preprocessing, involving the steps described below, was applied for consistent functional network topologies [100]: removal of the first 5 volumes to allow for steady-state magnetization,functional realignment, motion correction, and spatial normalization to the Montreal Neurological Institute (MNI-152 standard space with 2 × 2 × 2 mm isotropic resolution. A denoising procedure was driven by applying the anatomical CompCor (aCompCor method of removing cardiac and motion artifacts, by regressing out of each individual’s functional data the first 5 principal components corresponding to white matter signal, and the first 5 components corresponding to cerebrospinal fluid signal, as well as six subject-specific realignment parameters (three translations and three rotations and their first-order temporal derivatives [14]. The subject-specific denoised BOLD signal time series were first linearly detrended, and band-pass filtered between 0.008 and 0.09 Hz to eliminate both low-frequency drift effects and high-frequency noise. We did not apply spatial smoothing since all analyses were performed on parcellated data. The voxel-based time series within every ROI were averaged to extract a representative time series per ROI. In the present study, the AAL atlas [158] with 90 total brain areas (45 ROIs per hemisphere) was considered.

#### Static functional connectivity network construction: the multiplex way

**Fig. 1:**
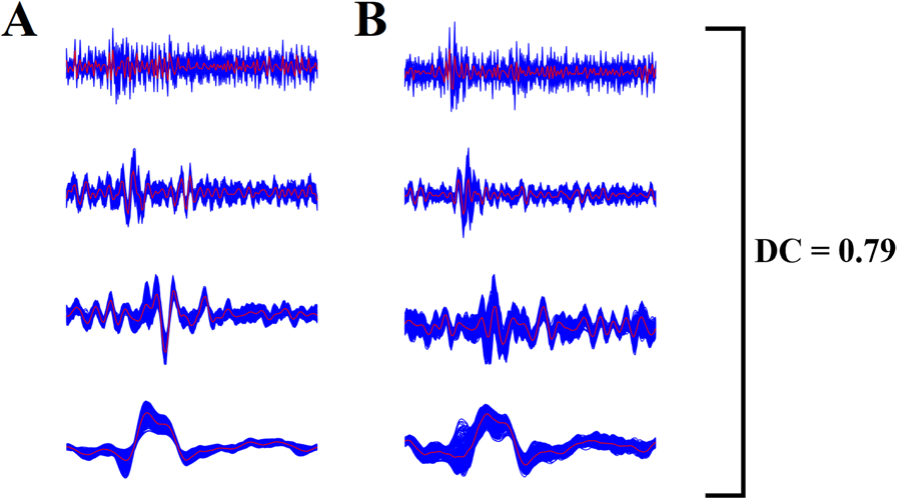
Wavelet decomposition of voxel-based time series and a multiplex coupling strength index. (A, B) A pair set of voxel-based time series (blue) was decomposed with the maximal overlap discrete wavelet transform (MODWT) in four frequency bands by adopting the Daubechies family implemented with a wavelet length equal to 6 [52]. The four wavelet scales, which correspond to the frequency ranges 0.125∼0.25 Hz (scale 1), 0.06∼0.125 Hz (scale 2), 0.03∼0.06 Hz (scale 3), and 0.015∼0.03 Hz (scale 4) [179].We then averaged the voxel-based time series producing four representative time series per frequency scale and per ROI (red). The distance correlation DC was estimated on the pair of four regional time series

In our study, the construction of a static functional connectivity network (sFCN) incorporates the wavelet decomposition of voxel-based time series and a distance correlation metric to quantify the multiplexity between two brain areas. We performed a wavelet decomposition on every voxel-based time series within every ROI by adopting the maximal overlap discrete wavelet transform (MODWT) selecting the Daubechies family implemented with a wavelet length equal to 6 [52]. Wavelet coefficients were extracted for the first four wavelet scales for every voxel-based time series which were further averaged to produce 4 frequency-dependent regional time series. The four wavelet scales, which correspond to the frequency ranges 0.125∼0.25 Hz (scale 1), 0.06∼0.125 Hz (scale 2), 0.03∼0.06 Hz (scale 3), and 0.015∼0.03 Hz (scale 4) [179]. Figure 1 illustrates this procedure for a pair of ROIs. Then, we adopted the distance correlation (DC) metric to quantify the multiplexity coupling strength between every pair of ROIs [147]. With the DC metric, one can estimate analytically the corresponding *p*-value of each coupling. This procedure leads to an sFCN of size 90 × 90 per subject and scans in both datasets.

#### Cognitive performance

We assessed cognition using a theoretically driven [94, 139] selection of tests that addressed the most relevant cognitive functions: Flexibility (Trail Making Test B-A time,[156], Fluency (letter and category fluencies,[114], Inhibition (interference—Stroop Test,Golden, 2001), Working Memory (backward—WAIS-III; [171], Visuospatial Function (copy accuracy—Rey-Osterrieth Complex Figure,[119], Language (Boston Naming Test-15,[71], Attention (forward span, digit symbol coding, and symbol search—WAIS-III,[171], Speed (Trail Making Test-A,[156],copy time—Rey-Osterrieth Complex Figure; [119], Visual Memory (memory accuracy—Rey Osterrieth Complex Figure,[119], and Verbal Memory (total learning and recall-II—Rey Auditory Verbal Learning Test,[129]. Six general domains were designed: (1) executive function, (2) visuospatial function, (3) language, (4) attention-speed, (5) memory, and (6) global cognitive function. The cognitive assessment was conducted before the CRF test or any type of exercise to control for acute exercise’s effect on cognitive performance. Extended details are in Supplementary Material Table 1.

#### Physical activity

We obtained physical activity levels with the Minnesota Leisure Time Physical Activity Questionnaire (VREM; [123]) in which frequency and duration during the last month of multiple activities — sportive walking, sport/dancing, gardening, climbing stairs, and shopping — are asked. Then, we calculated energy expenditure for each activity transforming hours per month into units of the metabolic equivalent of tasks (METs). We derived a measure of Sportive Physical Activity (S-PA) by adding METs spent in sportive walking and sport/dancing activities and a measure of Non-Sportive Physical Activity (NS-PA) by summing METs spent in gardening, climbing stairs, shopping, walking, and cleaning house.

#### Cardiorespiratory fitness

CRF was assessed using the Rockport 1-Mile Test and estimated the maximal aerobic capacity (VO_2max_) using the standard equation reported by Kline et al. [88]. The equation uses the following variables to estimate VO_2max_: weight, age, sex, time to complete a mile, and heart rate at the end of the test. During the test, participants were instructed to walk one mile on a treadmill adjusting their speed in order to be as fast as possible without running.

### Statistical analysis

Statistical procedures were performed using IBM SPSS Statistics 27. The distribution of raw scores was assessed to ensure data quality (i.e., outliers, skewness). We calculated change scores (post-test minus pre-test) and compared baseline scores between groups.

#### Detecting functional connectivity differences between pre- and post-intervention time periods

1. We applied ANOVA between the four groups global mean strength of sFCN to assess potential baseline differences. We also applied ANOVA between baseline and follow-up global mean strength of sFCN for the four groups. We adopted a similar approach between every pair of scans for the test–retest NYU dataset. The Kolmogorov–Smirnov test was used to check for normality of the data.
2. To reveal functional connectivity differences due to intervention, we applied a Wilcoxon Rank-Sum Test between pre- and post-intervention-related sFCN independently for every group and for every pair of ROIs. Finally, we corrected for multiple comparisons with a false discovery rate (FDR) with a significance level of *p* < 0.05 [16]. We followed a similar approach between every pair of scans for the test–retest NYU dataset.
3. To reveal functional connectivity differences in the baseline at ROI level between each intervention group and the control group (AE vs control; CCT vs control; COMB vs control), we adopted a Wilcoxon rank-sum test. Results were corrected for multiple comparisons using FDR, with a significant level of *p* < 0.05.

#### Assessing the repeatability of sFCN from the NYU dataset

4) We applied similar statistical analyses as described in (1) and (2) between the scan-based sFCN for the NYU dataset to assess the repeatability of the functional connectivity patterns as derived from the adopted pipeline.

#### Moderating and mediation analysis

5) We adopted the PROCESS Macro for SPSS [78] to analyze the moderating effect of age and sex on intervention-related changes for global mean DC strength estimated over the individual sFCN.
6) We also used the PROCESS macro to perform mediation analyses to assess whether a change in the global mean DC strength estimated over the individual sFCN mediated the cognitive benefits observed in the AE and COMB groups [121]. These benefits include for the AE group, the Executive Function (Working Memory) and Attention-Speed (Attention) and in the COMB group, the changes in Attention-Speed (Attention and Speed).
7) Complementarily, our mediation analysis was also driven by the outcome of the analysis over the sFCN difference maps (post-test minus pre-test) where the comparison between pre-post periods for each active group (AE-pre vs AE-post; CCT-pre vs CCT-post; COMB-pre vs COMB-post) revealed a subnetwork of functional connections that increased (positive network) or decreased (negative) due to the intervention. The mean DC strength of either the positive or negative subnetwork acted as mediators.
8) We also explored if the outcome of the analysis over the sFCN difference maps (post-test minus pre-test) mediated the relationship between the changes in the CRF and changes in the cognitive measures targeting on the Executive Function (Working Memory) and the Attention-Speed (Attention).

For these mediation analyses, we created a treatment variable (condition vs control) as the independent variable, change in cognition for those functions that showed significant intervention-related changes as the dependent variable, and changes in the CRF, the global mean DC strengths and the mean DC strength of the positive/negative subnetwork have been the mediators controlling for baseline performance score, age, sex, and years of education. These analyses were computed with bias-corrected bootstrapped 95% confidence intervals (CIs) based on 5000 bootstrap samples. The significance was indicated if the CIs in path AB did not overlap with 0 [78].

### Effect size and post hoc power estimation

An *effect size* is a value measuring the strength of the relationship between two variables in a population. Cohen’s *d* is defined as the difference between two means divided by a standard deviation for the data. Equation 1 illustrates mathematically the estimation of Cohen’s *d* effect size [37]:

1 $$d=\frac{\overline{{x }_{1}}-\overline{{x }_{2}}}{s}$$

Jacob Cohen defined *s*, the pooled standard deviation, as (for two independent samples):

2 $$s= \sqrt{\frac{\left({n}_{1}-1\right){s}_{1}^{2}- ({n}_{2}-1){s}_{2}^{2}}{{n}_{1}+{n}_{2}-2}}$$

where the variance for every intervention group in the baseline is defined as

3 $$s_1^2=\frac1{n_1-1}\sum\nolimits_{i=1}^{n_1}{(x_{i,1}-\overline{x_1})}^2$$

and similarly for every intervention group in the follow-up condition.

**Fig. 2:**
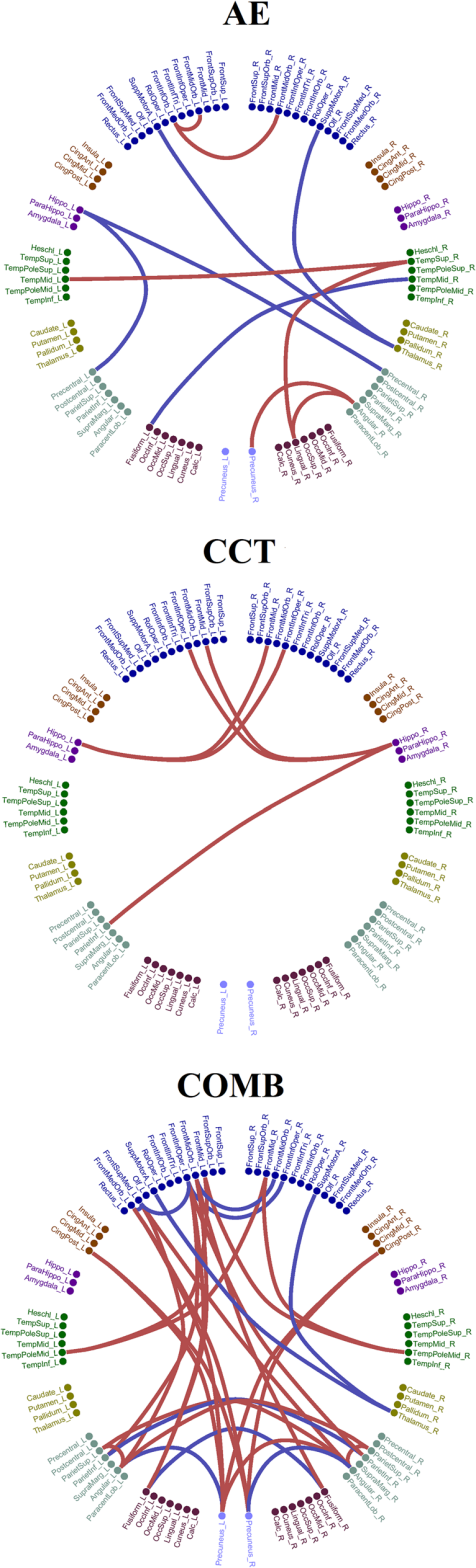
Topological layouts of increased (red) and decreased (blue) rsFC between pairs of ROIs in the AAL space for every intervention group comparing the baseline and the follow-up conditions. An interested reader can read this figure in conjunction with STable 4 in section 2 in the sup. material (red = increased coupling DC strength due to the intervention; blue = decreased coupling DC strength due to the intervention)

As means, we inputted to the formula the mean increment and decrement of DC strength over the detected functional pairs tabulated in Table 3 and shown in Fig. 2. It is important to mention here that a Cohen’s d higher than 0.8 is large, higher than 1.2 very large, and higher than 2 is huge. The higher the Cohen’s *d*, the lower the desirable sample size in order to get findings with high confidence and smaller alpha type I error. The alpha type I error refers to the probability to obtain a significant finding by chance. The adoptation of a false discovery rate (FDR) with a significance level of *p* < 0.05 in our rsFC analysis minimizes this chance. Here, as in the majority of studies, we set alpha = 0.05 which means that there is a 5% probability that a significant difference will occur by chance (false-positive). Β is the probability of not finding a difference when it actually exists (false-negative), and here it was set to *β* = 0.10. Power = 1 − *β* and expresses the ability to detect a difference when it exists. In our study, we set the Power = 0.90 [36].

Suppose the baseline and follow-up conditions are “A” and “B,” and we collect a sample from both scanning periods, i.e., we have two samples. We perform a two-sample test to determine whether the mean in group A, *μ*_A_, is different from the mean in group B, *μ*_B_. The hypotheses are

4 $${H}_{0}={\mu }_{\rm A}- {\mu }_{\rm B}=0 \& {H}_{1}={\mu }_{\rm A}- {\mu }_{\rm B}\ne 0$$

We computed the post hoc power according for a 2-sample 2-sided test adopting the equations reported in Chow et al. [36].

The post hoc power was estimated by the following set of equations:

5 $${n}_{A}=\kappa {n}_{B}\; \mathrm{ and}\; {n}_{B}=\left(1+ \frac{1}{\kappa }\right){\left(\sigma \frac{{z}_{1-\frac{a}{2}}+{z}_{1-\beta }}{{\mu }_{\rm A}-{\mu }_{\rm B}}\right)}^{2}$$

$$1-\beta =\Phi \left(z- {z}_{1-\frac{a}{2}}\right)+ \Phi \left(-z- {z}_{1-\frac{a}{2}}\right), z=\frac{{\mu }_{\rm A}-{\mu }_{\rm B}}{\sigma \sqrt{\frac{1}{{n}_{A}}+\frac{1}{{n}_{B}}}}$$

where

*κ* = *n*_A_/*n*_B_: is the matching ratio, and *n*_A_ and *n*_B_ are the actual sample sizes of the group
*σ*: is standard deviation
Φ: is the standard Normal distribution function
Φ^−1^: is the standard Normal quantile function
*α*: is type I error
*β*: is type II error, meaning 1 − *β* is power

Both Cohen’s *d* and post hoc power were estimated for the averaged DC strength over the pairs of ROIs shown in Fig. 2 independently for the increment and decrement groups (Table 3).

## Results

### Participants

A total of 109 participants completed the baseline assessment and 92 completed the intervention (intention to treat sample, ITT) (see Figure 1 in Roig-Coll et al. [121]. The Per Protocol (PP) sample, analyzed in the present study, included 82 subjects (62% female,age = 58.38 ± 5.47) with a level of adherence > 80%. The demographics of the PP sample are tabulated in Table 2. There were no participants’ differences at the baseline across the groups in physical and cognitive outcomes except for Non-Sportive Physical Activity (NS-PA) and current smoking status (see supp. material Tables 2.1, 2.2 for extended details). Current smoking status at baseline was included as a covariate. We applied Kolmogorov–Smirnov tests for the normality of the demographic data showed in Table 2 and also in STables 2.1, 2.2. In variables where the normality was supported, we applied ANOVA; otherwise, we adopted the Kruskal–Wallis test.

**Table 2:** Participants characteristics at baseline

	Total Mean (*SD*)	AE Mean (*SD*)	CCT Mean (*SD*)	COMB Mean (*SD*)	Control Mean (*SD*)	Group comparison
*n* total / *n* females	82 / 51	25 / 13	23 / 16	19 / 14	15 /8	*Χ*^2^(3) = 3.20, *p* = .361
Age (years)	58.38 (5.47)	58.40 (5.12)	57.91 (5.31)	60.32 (5.54)	56.60 (5.97)	*H*(3) = 3.53, *p* = .317
Years of education	12.52 (5.57)	12.44 (5.75)	12.04 (4.94)	12.37 (5.43)	13.60 (6.72)	*H*(3) = 0.28, *p* = .963

Mean (*SD*). See Supplementary Tables 4 for more cognitive, physical, cardiovascular risk factors, and global FA and MD outcomes at baseline

*AE* aerobic exercise; *BMI* body mass index; *CCT* computerized cognitive training; *COMB* combined training; *WAIS-III* Wechsler Adult Intelligence Scale, *X*^*2*^ chi-square; *H* Kruskal–Wallis *H* test; *F* ANOVA test

### Repeatable sFCN produced by the adopted pipeline

Our analytic pipeline revealed consistent mean functional connectivity coupling strength between short-term (mins) and long-term (months) scans as estimated over the rs-fMRI test–retest NYU dataset (see supp. Table 2.3). Moreover, we did not detect any significant difference between every pair of scans on the ROI-ROI level. Both analyses, on the global and local levels, favor the adoptation of the pipeline for the analysis of the rs-fMRI recordings from our intervention study.

### Intervention-related changes in static functional connectivity

The global mean strength of sFCN did not differ significantly between the four groups at the baseline (see supp. material Table 2.3). Moreover, the global mean strength of the sFCN for the three groups (AE, CCT, and control) did not differ significantly between the baseline and the follow-up but it differs only for the COMB group (see supp. material Table 3).

Regarding the pre-post group analysis on the ROI level, Fig. 2 illustrates the significant connections that survived the statistical thresholds for every intervention group comparing the baseline and the follow-up conditions. The AE and CCT interventions led to a small number of connections, 11 and 6 connections, correspondingly while the COMB intervention protocol produced an extended network of 33 connections (Fig. 2). Our findings involved a combination of increased coupling DC strength (red) and decreased coupling DC strength (blue) due to the intervention protocol. The averaged increment and decrement of DC strength over the detected pairs of connections are tabulated in Table 3 showing no group difference.

**Table 3:** Averaged increment and decrement of DC strength over the detected pairs of connections demonstrated in Fig. 2

	AE *M*(*SD*) increased–*M*(*SD*) decreased	CCT *M*(*SD*) increased–*M*(*SD*) decreased	COMB *M*(*SD*) increased–*M*(*SD*) decreased
Mean DC strength (Moviment)	0.035 (0.005)–0.021 (0.007)	0.031 (0.006)	0.041 (0.006)–0.032 (0.005)

*AE* aerobic exercise, *CCT* computerized cognitive training, *COMB* combined training, *M* mean, *SD* standard deviation

Specifically, for the AE group, we revealed an increased rsFC between the right lingual gyrus (Visual Network — VN) and right superior temporal gyrus (ventral attentional network — VAN), and the right angular gyrus (fronto-parietal network — FPN), between the right superior temporal gyrus (ventral attentional network — VAN), and left middle temporal gyrus (posterior DMN — pDMN), between the right angular gyrus (VN) and right precuneus (pDMN), and between the left and right inferior frontal gyri (orbital part) with left frontal middle gyrus (orbital part) (anterior DMN — aDMN). We also revealed a decreased rsFC between the left fusiform gyrus (pDMN) and right middle temporal gyrus (pDMN), between the left hippocampus and left and right precentral gyrus (pDMN), and between the left and right supplementary motor areas and the right thalamus.

For the CCT group, we untangled an increased rsFC between the left hippocampus (pDMN) with the right inferior frontal gyrus (opercular part) (aDMN) and the right middle frontal gyrus (aDMN), between the right hippocampus (pDMN) with the left middle frontal gyrus (aDMN), and the left inferior frontal gyrus (opercular part) (aDMN), and between the left superior frontal gyrus (aDMN), and the left inferior parietal gyrus (pDMN).

The strongest changes were observed in the COMB group in brain areas that involved an increased rsFC of within FPN coupling strength between the left and right middle frontal gyri and the left and right temporal poles (middle temporal gyri), the increment between the left and right superior parietal lobules and the left and right angular gyri (FPN), an increased rsFC between the left and right middle frontal gyri (FPN) and the left and right temporal poles (middle temporal gyri), the increment between the posterior DMN and FPN that involved the left/right precuneus (pDMN) with the right angular gyrus (FPN) and with the posterior cingulate gyrus (pDMN), and an increased rsFC between the left portion of the aDMN that involves the left middle frontal gyrus (orbital part), the inferior frontal gyrus (opercular part), the superior frontal gyrus (medial part), and the middle frontal gyrus (orbital part) with the pDMN that involves the left/right fusiform gyrus, the left/right parietal inferior gyri, the left/right angular gyrus, and the left/right precuneus. The COMB intervention group showed a decreased rsFC between areas within the pDMN (left and right fusiform gyri, left and right inferior parietal gyri, left angular gyrus-left precuneus, right angular-right precuneus), within the aDMN (left and right middle frontal gyri (orbital part), left and right inferior frontal gyri-opercular part, left inferior frontal gyrus (opercular part) with the left superior frontal gyrus (medial part)), between the left superior frontal gyrus (medial part) with the left middle frontal gyrus (orbital part) and between the left and right supplementary motor with the right thalamus.

The mean and total DC coupling strength of the detected connections within aDMN (decrement), within pDMN (decrement), and between aDMN and pDMN (increment) brain areas will also feed the mediation analysis (see the “Mediation effects on intervention-related cognitive benefits” section).

Statistical analysis of sFCN in a pairwise ROI fashion between the intervention group and the control in the baseline (AE vs control; CCT vs control; COMB vs control) did not reveal any findings.

### *Estimated effect size and *post hoc* power estimation*

Based on known sample size, the power in the three groups and in both groups of functional pairs of ROIs that showed either increment or decrement with the baseline was above 0.90 for all cases. The Cohen’s *d* effect size was between 0.78 and 1.13 which means that our findings showed between large and very large effect size (Table 4).

**Table 4:** Cohen’s *d* effect size and power estimations estimated independently over the averaged increment and decrement of DC strength of the detected pairs of connections demonstrated in Fig. 2

	AE Increased–decreased	CCT Increased	COMB Increased–decreased
Cohen’s *d*/power (Moviment)	1.13/1–0.78/0.96	0.84/1	0.81/1–1.05/1

*AE* aerobic exercise, *CCT* computerized cognitive training, *COMB* combined training, *M* mean, *SD* standard deviation

### Sex and age moderation effects

Moderation analyses showed that age did not significantly moderate the effects of the intervention on global mean rsFC strength estimated over the individual sFCN in any group. Sex did not significantly moderate the effects of the intervention on global mean rsFC strength estimated over the individual sFCN in any group.

### Mediation effects on intervention-related cognitive benefits

We applied mediation analyses to investigate whether changes in rsFC mediated the association between the intervention and the cognitive domains that demonstrated a significant change as reported in Roig-Coll et al. [121]. As rs-FC, we employed the global mean rsFC strengths, the mean, and the total rsFC strength of the positive/negative subnetworks that involved the within aDMN, the within pDMN, and their interactions (aDMN-pDMN). In our previous study, we showed that the AE group showed improvement in Executive Function (Working Memory) and Attention-Speed (Attention) and the COMB group showed changes in Attention-Speed (Attention and Speed). Mediation analyses showed that changes in global mean DC strengths and mean and total DC strength of the positive/negative subnetwork did not significantly mediate the observed cognitive benefits for any group. Finally, the integrated increment and decrement of rsFC changes detected (Fig. 2) did not mediate the relationship between CRF and executive Function (Working Memory) and Attention-Speed (Attention).

## Discussion

In this paper, we report intervention-related changes in rs-fMRI static functional connectivity in our Projecte Moviment trial, which investigates the potential neuroprotective effects of interventions (AE, CCT, COMB) in healthy physically inactive late-middle-aged adults compared to a healthy control group [29]. In our previous study, we reported cognitive changes in Executive Function and Attention-Speed in the AE group, and Attention-Speed in the COMB group [121].

In our study, the participants who followed the AE program showed positive changes in rs-fMRI functional connectivity in agreement with previous studies [142, 143]. We reported an increased rsFC that involved mainly the aDMN, pDMN, and also FPN, VAN, and VN, and negative rsFC changes that involved the pDMN, the hippocampus, the left and right supplementary motor areas and the right thalamus. Previous studies reported an association between AE and increased coupling within the DMN [35, 106], in the hippocampal network [175] and also a decreased rsFC within the DMN [35, 105], and the SMN [63]. Although the interpretation of the decreased rsFC is challenging, AE-related decreased rsFC co-occurred with decreased fat mass [105] and cognitive stability [35]. It is evident from our findings that longer interventions are needed to induce extended changes in both metabolic and rs-fMRI connectivity networks. Parameters of the AE are critical for the neuroprotective effects of the exercise which can be altered by sex, age, and health status [144, 175].

For the CCT group, we reported an increased rsFC between the left hippocampus (pDMN) with the right inferior frontal gyrus (opercular part) (aDMN) and the right middle frontal gyrus (aDMN), between the right hippocampus (pDMN) with the left middle frontal gyrus (aDMN), and the left inferior frontal gyrus (opercular part) (aDMN), and between the left superior frontal gyrus (aDMN), with the left inferior parietal gyrus (pDMN). Previous studies in healthy older adults reported a combination of increased rsFC and decreased rsFC in the DMN as a consequence of a CCT program (for a review, see Ten Brinke et al. [154]. The maintenance of global mean rsFC on the same level in conjunction with an increased rsFC pattern that involved DMN and also the hippocampus could be characterized as a positive outcome of CCT intervention. These observations could be linked not only to the CCT protocol but also to the general motivation of the subjects that participated in a lifestyle behavior project. In our previous study [121], participants in the CCT did not show any significant changes in physical activity status, sleep patterns, and psychological health.

Our study also evaluates the benefits of combining multimodal CCT and brisk walking for 12 weeks, 5 days per week in bouts of 54 min. Participants of the COMB group showed an improvement in both global and local rsFC compared to the control group. Our findings supported both the benefits of COMB intervention in terms of both local and global rsFC. The increment of global rsFC is a positive outcome of the COMB intervention by comparing the global mean of our group of old adults compared to the global mean of younger adults from the NYU dataset (see supp. material Table 2.3 for NYU dataset vs supp. material Table 3). Our pre-post analysis of rsFC in the COMB group revealed an extended network of functional connections where their strength either increased or decreased in the follow-up compared to the baseline. This network involved mainly the DMN and to a lesser extent the FPN and also the supplementary motor area with the thalamus as it was observed in the AE group. Specifically, we observed a decreased rsFC pattern for brain areas located within the aDMN and within the pDMN and an increased rsFC pattern between brain areas located over the aDMN and the pDMN. A reduced rsFC was also detected between the left and right supplementary motor areas and the right thalamus. Our rsFC findings supported the positive outcome of the COMB intervention protocol within the 12 weeks period compared to the rsFC findings in CCT and AE groups. There is no rs-fMRI intervention study that followed the same protocol with three active and a control group in order to directly compare our findings.

DMN are defined as sets of anatomically distance brain areas that showed temporal correlations of their spontaneous resting-state fluctuations which is called functional connectivity [46]. The observed functional connectivity between remote brain areas with the use of resting-state fMRI is consistent with their anatomical connectivity as revealed using diffusion tensor imaging [68, 137]. Previous findings suggested that the functional connectivity strength between the DMN areas can partially be explained on white matter tracts namely the structural connectivity strength [152, 160, 161].

Despite the growing amount of accumulated knowledge regarding the physiology and anatomy of DMN, the main cognitive function of this network is still poorly understood. Different parts of DMN are involved in different high-level cognitive functions. For example, the posterior cingulate cortex (PCC) is active during tasks that are associated with autobiographical episodic memory and self-referential processes [130], the medial prefrontal cortex’s activity is linked to social cognitive processes [1], the medial temporal lobe is mainly active in episodic memory [164], and the inferior parietal cortex’s activity, and especially the angular gyrus, is engaged in semantic processing and attention [18].

The DMN consists of cortical brain areas that are anatomically distant from input to output systems of the brain in visual and motor cortex. Previous findings suggest that the DMN consists of brain areas that are on the top of a representational hierarchy that involves the cognitive landscape of abstract terms. These terms revealed by a meta-analysis of published research articles are the “social cognition,” “verbal semantics,” and “autobiographical memory”—tasks that rely on complex representations abstracted away from specific sensory and motor processes [104]. The topological location of the DMN brain areas that are geodesic distance from input–output systems of the brain may support the expression of stimulus independent aspects of cognitive functions associated with mind-wandering [23]. The DMN is also implicated in specific domains of cognition that are critical during mind-wandering, including social cognition, semantic and episodic memory, and future planning (for meta-analyses, see Spreng et al. [140] and Andrews-Hanna et al. [3].

Mean rsFC in the whole-brain and within the DMN showed a nonlinear trajectory in healthy older adults with more rapid declines in older age and a possible increment in early stages of the aging process (Staffaroni et al., 2018). This study investigated how rsFC changes within the age spectrum (55–90 age) in normal aging in three DMN subnetworks: (1) within-DMN, (2) between anterior and posterior DMN, and (3) between medial temporal lobe network and posterior DMN. The partition of DMN in anterior and posterior parts has been previously reported in the literature [44]. In this longitudinal study, they revealed that rsFC within-DMN and between anterior and posterior regions of the DMN predicted changes in memory performance (Staffaroni et al., 2018). However, rsFC showed significant age × time interaction effect only for the whole-brain and the within DMN areas but not with anterior–posterior and between medial temporal lobe and posterior DMN.

RsFC within the DMN is the most common resting-state network under investigation that has been also found to be impacted by aging [66, 107]. In normal aging, various DMN areas like the superior and middle frontal gyrus, superior parietal cortex, and posterior cingulate cortex showed a decreased rsFC [74]. Age-associated changes in rsFC were also observed between the anterior versus the posterior DMN [2]. In the aDMN, both increment and decrement were revealed in the frontal lobe, whereas within the pDMN, only a decreased rsFC pattern was detected associated with aging [169]. It is hypothesized that the increased rsFC in the aDMN could serve as a compensatory mechanism that attempts to balance the loss of cognitive functions [73, 169]. It is the first time in the literature that an intervention study reports changes in rsFC within aDMN, pDMN, and also between aDMN and pDMN brain areas.

Neuroimaging findings from various task-evoked activity studies have isolated brain areas of the dorsal frontal and posterior parietal cortex to be involved in the direction of attention to spatial locations [39, 109]. These brain areas form the dorsal attention system (DAN) [89] which maintain endogenous signals linked to goals relevant to top-down modulatory signals biasing further the stimulus processing [21]. A secondary attention system is the ventral attention system (VAN) which is located in the temporo-parietal junction and the ventral frontal cortex (Corbetta et al., 2000), and it was assigned with the functional role of directing attention to salient sensory events [64]. These networks could be investigated even in the absence of tasks such as the spontaneous resting-state activity (Fox et al., 206). For daily human activities, and especially for older adults, it is essential to orient attention to behaviorally relevant stimuli which mainly involves the brain activity of the dorsal and ventral fronto-parietal networks (FPN) (Arif et al., 2020). Age modulates FPN during attentional reorienting demanding tasks. This study showed that older healthy adults were slower in reorienting attention and exhibited age-related alterations within parietal and frontal regions, which may reflect increased task demands depleting their compensatory resources. The VAN network is mostly linked to the temporo-parietal junction and ventral frontal cortices and is thought to be involved in bottom-up processes that are stimulus driven [135]. An increment of DC connectivity strength was revealed within the VAN network in the AE group.

Brain networks involved in primary information processing such as the visual network (VN) showed within-network connectivity significant positive association with older age [180]. Here, we untangled an increment within the VN for the AE group. It is important to stress here that aging causes both increments and decrements between well-known resting-state networks while the brain organization undergoes reorganization [180]. It is important to study how normal aging influences within and between networks connections, how these connectivity patterns altered in neurodegenerative diseases, and how non-pharmaceutical interventions like aerobic exercise can act as a protective mechanism against these diseases (Wang et al., 2021; [98]. The protective role of combined intervention protocols (aerobic exercise and cognitive training), against neurodegenerative diseases like the one proposed here, should be further evaluated [125].

In moderation analyses, we did not find a significant moderation effect of age on intervention-related changes in the global mean rsFC strength estimated over the individual sFCN in any group*.* These findings could be supported by the tight age-range of our sample and the fact that our sample was young compared to targeted clinical populations where the brain health of older subjects tends to decline faster compared to the intervention group [56]. We did not find a significant moderation effect of sex in the global mean rsFC strength estimated over the individual sFCN in any group. However, more studies from our group and other research groups suggested that biological age acts as a moderator of the relationship between aerobic exercise and neuroprotective effects [10, 11, 27–29]. Sex differences in the response of cardiovascular, musculoskeletal, and respiratory systems in AE and the impact of sex hormones may explain the different intervention-related changes in cardiovascular risk factors [10, 11].

To reveal the possible mechanisms that could explain the cognitive benefits in the AE group in the Executive Function (Working Memory) and Attention-Speed (Attention) and in the COMB group in the Attention-Speed (Attention and Speed) [121], we explored the mediation effects of the global mean rsFC strength, and the local rsFC findings in the COMB group (the mean rsFC strength within aDMN, within pDMN, and between aDMN and pDMN). No global and especially local rsFC changes due to any intervention mediated the cognitive benefits detected in the AE and COMB groups. Additionally, the integrated increment and decrement rsFC changes due to the intervention protocol (Fig. 2) did not mediate the relationship between CRF and the executive Function (Working Memory) and Attention-Speed (Attention) measurements.

The failure of the mediation analysis with rsFC as actual mediators could be explained due to the short duration of the intervention and/or the sample size which might not be enough to reveal such mediation effects in the cognitive functions of high variability [142].

While there is partial consistency in neuropsychological, and structural neuroimaging findings across the studies, results based on rsFC analysis are less consistent. The reason for this inconsistency is due to a large repertoire of available analytic paths that one researcher can follow. Below, we summarize the available rsFC options: the seed-based analysis targeting one or a few seed ROIs [35, 63, 106, 116, 175, 176], the ROI-ROI based on the parcellated space by adopting an atlas [165], the BOLD fluctuation [157], the graph theory methods [25, 157], and the application of ICA to detect the seven resting-state networks where this approach is called network-of-interest (NOI) [105]. The diversity of methodological analytic approaches across those reports is due to an explosion of the proposed alternative methods that one researcher can follow to investigate the rsFC. For that reason, differences between the analytic methods adopted by intervention studies should be taken into consideration in the final interpretation of the converging findings. Specifically, in our study which is the first rs-fMRI study that includes three active groups and a control group, our findings should be replicated by another study following a similar analytic approach. However, to guarantee that our findings are not spurious due to the adopted analytic plan that includes specific network construction steps [100], we ran the same analysis in a test–retest rs-fMRI study with three scans. The consistency of rsFC between scans in the NYU test-retest dataset further supports our analytic pipeline and the findings in the Projecte Moviment trial.

## Limitations

In our study, we revealed altered functional connectivity patterns in the three groups due to the intervention protocol. Our findings can be summarized in an increased and decreased rsFC pattern which was also supported by power analysis (Fig. 2). The findings were also supported also by the post hoc power analysis and the Cohen’s *d* effect size. However, it should be underlined that a larger number of participants would further advance our findings. Future intervention research should invite a larger number of participants followed by age and sex-balanced groups that will support intra-group comparative analyses. The adopted analytic pathway included a static functional connectivity analysis. It would be very important to analyze the same dataset following a dynamic functional connectivity analysis [50]. It would be interesting to also register diet patterns that influence cognitive functions and rsFC [51] and can potentially positively affect cardiovascular risk factors and anthropometric and blood sample measures. Additionally, it would be interesting to register the participants longitudinally at more than two-time points. Our Projecte Moviment will advance our understanding of the intervention protocols by taking the advantage of new omics technology that will further shed light on biological pathways supporting the cognitive benefits as a consequence of the intervention protocol.

## Conclusions

In summary, the present study demonstrates the potential benefits of lifestyle interventions when a combined physical and cognitive intervention protocol is adopted. For the AE group, we reported an increased rsFC that involved mainly the aDMN, pDMN, and also FPN, VAN, and VN and a decreased rsFC that involved the pDMN, the hippocampus, and the left and right supplementary motor areas, as well as the right thalamus. For the CCT group, we found a combination of increased and decreased rsFC between brain areas located mainly in the aDMN and pDMN. The greatest alterations of rsFC were revealed in the COMB group. These findings involved a decreased rsFC within the aDMN and within the pDMN, an increased rsFC between the aDMN and pDMN, and a reduced rsFC between the left and right supplementary motor areas and the right thalamus as it was found in the AE group.

### Supplementary Information

Below is the link to the electronic supplementary material.

Supplementary file1 (DOCX 2171 KB)

## Data Availability

The data that support the findings of this study are available from the corresponding author, [MM], upon reasonable request.
