# Supplementary material for: Intrinsic functional brain connectivity changes following aerobic exercise, computerized cognitive training, and their combination in physically inactive healthy late-middle-aged adults: the Projecte Moviment
Source: GeroScience. 2023 Oct 23;46(1):573–96. doi: 10.1007/s11357-023-00946-8 (PMC10828336; doi:10.1007/s11357-023-00946-8)
Supplement: Supplementary file 1 — Supplementary file1 (DOCX 2171 KB) [file 11357_2023_946_MOESM1_ESM.docx]

***Intrinsic functional brain connectivity changes following aerobic exercise, computerized cognitive training and their combination in healthy late-middle-aged adults: the Projecte Moviment***

**Stavros I. Dimitriadis^1,2^_,_ Alba Castells-Sánchez^1,2^, Francesca Roig-Coll^1,2^, Rosalía Dacosta-Aguayo^1,3,4^ , Noemí Lamonja-Vicente^1,2,3,5^, Pere Torán-Monserrat^3,6^, Alberto García-Molina^4,7^, Gemma Monte-Rubio^8^, Chelsea Stillman^9^, Alexandre Perera - Lluna^10,11^ and Maria Mataró^1,2,5^**

^1^Department of Clinical Psychology and Psychobiology, University of Barcelona, Barcelona, Spain

^2^Institut de Neurociències, University of Barcelona, Barcelona, Spain

^3^Unitat de Suport a la Recerca Metropolitana Nord, Fundació Institut Universitari per a la recerca a l’Atenció Primària de Salut Jordi Gol i Gurina, Mataró, Spain

^4^Institut d’Investigació en Ciències de la Salut Germans Trias i Pujol (IGTP), Badalona, Spain

^5^Institut de Recerca Sant Joan de Déu, Esplugues de Llobregat, Spain

^6^Department of Medicine, Universitat de Girona, Girona, Spain

^7^Institut Guttmann, Institut Universitari de Neurorehabilitació, Universitat Autònoma de Barcelona, Badalona, Spain

^8^ Centre for Comparative Medicine and Bioimage (CMCiB), Germans Trias i Pujol Research Institute (IGTP), Badalona, Spain

^9^ Department of Psychology, University of Pittsburgh, Pittsburgh, PA, United States.

^10^B2SLab, Departament d'Enginyeria de Sistemes, Automàtica i Informàtica Industrial, Universitat Politècnica de Catalunya, CIBER-BBN, Barcelona 08028, Spain.

^11^Department of Biomedical Engineering, Institut de Recerca Pediàtrica Hospital Sant Joan de Déu, Esplugues de Llobregat, Barcelona 08950, Spain.

*** Correspondence:**

**Maria Mataró, mmataro@ub.edu, (+34) 933125052**

**Address: Department of Clinical Psychology and psychobiology, University of Barcelona, Passeig Vall d’Hebron 171, 08035 Barcelona, Spain.**

**Section 1. Supplementary Tables**

| **Table 1**  Cognitive outcomes: variables and measures | | | |
| --- | --- | --- | --- |
| Composites 1^st^ Level | Composites 2^nd^ Level | Tests - Subtest | Measure |
| Executive Function | Flexibility | TMT B -A | Z score |
|  | Fluency | Letter fluency | Z score |
|  |  | Category fluency | Z score |
|  | Inhibition | Stroop - Interference | Z score |
|  | Working Memory | WAIS III - Backward Span | Z score |
| Visuospatial Function | Visuospatial Function | ROCF - Copy Accuracy | Z score |
| Language | Language | BNT (15 items) | Z score |
| Attention - Speed | Attention | WAIS III - Forward Span | Z score |
|  |  | WAIS III - Digit Symbol Coding | Z score |
|  |  | WAIS-III - Symbol Search | Z score |
|  | Speed | TMT - A | Z score |
|  |  | ROCF - Copy Time | Z score |
| Memory | Visual Memory | ROCF - Memory Accuracy | Z score |
|  | Verbal Memory | RAVLT - Total Learning | Z score |
|  |  | RAVLT - Recall II | Z score |
| Global Cognitive Function | *Sum of all domains* | | Z score |
| BNT, Boston Naming Test (Goodglass et al., 2001); RAVLT, Rey Auditory Verbal Learning Test (Schmidt, 1996); ROCF, Rey-Osterrieth Complex Figure (Rey, 2009); Stroop Test (Golden, 2001); TMT, Trail Making Test (Tombaugh, 2004); Verbal Fluency Tests (Peña-Casanova et al., 2009); WAIS-III, Wechsler Adult Intelligence Scale (Wechsler, 2001). | | | |

| **Table 2.1**  Group comparison at Baseline: z-scores of Cognitive Domains | | | | | |
| --- | --- | --- | --- | --- | --- |
| Variables | Groups | n | Mean | SD | ANOVA / H de Kruskall Wallis |
|  |  |  |  |  |  |
| Executive Function | AE | 24 | -0.01 | 0.72 | F(3,75) = 0.81, *p* = .492 |
|  | CCT | 23 | 0.16 | 0.60 |  |
|  | COMB | 19 | -0.08 | 0.50 |  |
|  | Control | 13 | -0.15 | 0.81 |  |
| Flexibility | AE | 25 | 0.10 | 1.05 | H(3) = 1.53, *p* = .676 |
|  | CCT | 23 | -0.10 | 1.06 |  |
|  | COMB | 19 | -0.03 | 0.80 |  |
|  | Control | 14 | 0.04 | 1.12 |  |
| Fluency | AE | 25 | 0.02 | 0.91 | F(3,77) = 1.19, *p* = .318 |
|  | CCT | 23 | 0.22 | 0.76 |  |
|  | COMB | 19 | -0.10 | 0.82 |  |
|  | Control | 14 | -0.28 | 0.80 |  |
| Inhibition | AE | 24 | -0.06 | 1.03 | F(3,77) = 1.18, *p* = .323 |
|  | CCT | 23 | 0.32 | 0.86 |  |
|  | COMB | 19 | -0.16 | 1.18 |  |
|  | Control | 15 | -0.20 | 0.89 |  |
| Working Memory | AE | 25 | -0.06 | 1.10 | H(3) = 1.29, *p* = .732 |
|  | CCT | 23 | 0.14 | 1.05 |  |
|  | COMB | 19 | -0.01 | 0.69 |  |
|  | Control | 15 | -0.11 | 1.16 |  |
| Visuospatial Function | AE | 25 | 0.14 | 0.93 | H(3) = 3.40, *p* = .334 |
|  | CCT | 23 | -0.20 | 1.04 |  |
|  | COMB | 19 | -0.19 | 1.24 |  |
|  | Control | 15 | 0.32 | 0.62 |  |
| Language | AE | 25 | 0.01 | 1.09 | H(3) = 0.71, *p* = .870 |
|  | CCT | 23 | -0.04 | 1.08 |  |
|  | COMB | 19 | -0.10 | 0.91 |  |
|  | Control | 15 | 0.16 | 0.89 |  |
| Attention-Speed | AE | 24 | -0.02 | 1.01 | H(3) = 2.34, *p* = .506 |
|  | CCT | 23 | 0.13 | 0.65 |  |
|  | COMB | 19 | -0.17 | 0.68 |  |
|  | Control | 14 | 0.09 | 0.44 |  |
| Attention | AE | 25 | 0.03 | 0.95 | F(3,77) = 0.70, *p* = .553 |
|  | CCT | 23 | 0.15 | 0.71 |  |
|  | COMB | 19 | -0.20 | 0.76 |  |
|  | Control | 14 | 0.03 | 0.62 |  |
| Speed | AE | 24 | -0.12 | 1.21 | H(3) = 1.64, *p* = .650 |
|  | CCT | 23 | 0.11 | 0.77 |  |
|  | COMB | 19 | -0.12 | 0.74 |  |
|  | Control | 15 | 0.17 | 0.32 |  |
| Memory | AE | 25 | 0.03 | 0.70 | H(3) = 0.95, *p* = .815 |
|  | CCT | 23 | 0.13 | 0.66 |  |
|  | COMB | 19 | -0.14 | 0.92 |  |
|  | Control | 14 | -0.09 | 0.95 |  |
| Visual Memory | AE | 25 | 0.17 | 1.05 | F(3,78) = 1.00, *p* =.398 |
|  | CCT | 23 | 0.06 | 0.98 |  |
|  | COMB | 19 | -0.34 | 1.17 |  |
|  | Control | 15 | 0.04 | 0.65 |  |
| Verbal Memory | AE | 25 | -0.04 | 0.71 | H(3) = 0.70, *p* = .874 |
|  | CCT | 23 | 0.16 | 0.79 |  |
|  | COMB | 19 | -0.04 | 1.08 |  |
|  | Control | 14 | -0.14 | 1.29 |  |
| Global Cognitive Function | AE | 23 | 0.01 | 0.73 | F(3,74) = 0.51, *p* = .675 |
|  | CCT | 23 | .011 | 0.56 |  |
|  | COMB | 19 | -0.13 | 0.57 |  |
|  | Control | 13 | -0.03 | 0.59 |  |
| AE = Aerobic exercise; CCT = Computerized Cognitive Training; COMB = Combined Training. **F = Anova test ; H = Kruskall Wallis H test** | | | | | |

| **Table 2.2**  Group Comparison at Baseline: PA and CRF | | | | | |
| --- | --- | --- | --- | --- | --- |
| Variables | Groups | N | Mean | SD | ANOVA / H de Kruskall Wallis |
| CRF | AE | 19 | 25.25 | 10.16 | F(3,67) = 1.08, *p* = .362 |
|  | CCT | 20 | 26.11 | 12.50 |  |
|  | COMB | 17 | 27.34 | 8.75 |  |
|  | Control | 15 | 20.65 | 12.69 |  |
| S-PA | AE | 25 | 451.98 | 699.40 | H(3) = 2.92, *p* = .404 |
|  | CCT | 23 | 439.83 | 713.63 |  |
|  | COMB | 19 | 778.79 | 908.77 |  |
|  | Control | 15 | 366.80 | 618.17 |  |
| NS-PA | AE | 25 | 5595.73 | 3918.34 | H(3) = 7.96, *p* = .047* |
|  | CCT | 23 | 9113.74 | 7104.64 |  |
|  | COMB | 19 | 10295.68 | 6159.04 |  |
|  | Control | 15 | 7038.40 | 6628.45 |  |
| AE = Aerobic exercise; CCT = Computerized Cognitive Training; COMB = Combined Training; CRF = Cardiorespiratory Fitness; NS-PA = Non Sportive Physical Activity; S-PA = Sportive Physical Activity; Total-PA = Total Physical Activity.  **F = Anova test ; H = Kruskall Wallis H test**  **p* < 0.05 | | | | | |

| **Table 2.3**  Group Comparison at Baseline: Global Mean DC Strength estimated over the individual sFCN for the MOVIMENT PROJECTE and the NYU test-retest study. Kolmogorov-Smirnov tests supported the normality of the data and the use of ANOVA. | | | | | |
| --- | --- | --- | --- | --- | --- |
| Variables | Groups | N | Mean | SD | ANOVA / H de Kruskall Wallis |
| Global Mean DC Strength (MOVIMENT) | AE | 25 | 0.179 | 0.0052 | F(3,78) = 2.04, *p* = .125 |
|  | CCT | 23 | 0.176 | 0.0060 |  |
|  | COMB | 19 | 0.175 | 0.0050 |  |
|  | Control | 15 | 0.177 | 0.0060 |  |
| Global Mean DC Strength (NYU) | SCAN1 | 25 | 0.2060 | 0.002 | F(3,74) = 1.37, *p* = .259 |
|  | SCAN2 | 25 | 0.2070 | 0.002 |  |
|  | SCAN3 | 25 | 0.2060 | 0.002 |  |
|  |  |  |  |  |  |
| AE = Aerobic exercise; CCT = Computerized Cognitive Training; COMB = Combined Training; FA = Fractional Anisotropy; MD = Mean Diffusivity ;  **F = Anova test** | | | | | |

| **Table 3**  Comparison between baseline and follow-up intragroup: Global Mean DC Strength estimated over the individual sFCN. Kolmogorov-Smirnov tests supported the normality of the data and the use of ANOVA. | | | | |
| --- | --- | --- | --- | --- |
|  | **AE**  M(SD) Baseline - M(SD) Follow-up  t(d.f.); *p* Value | **CCT**  M(SD) Baseline - M(SD) Follow-up  t(d.f.); *p* Value | **COMB**  M(SD) Baseline - M(SD) Follow-up  t(d.f.); *p* Value | **Control**  (SD) Baseline - M(SD) Follow-up  t(d.f.); *p* Value |
| Global Mean DC Strength (MOVIMENT) | 0.179 (0.005) – 0.181 (0.008)  t(24)=2.88; *p*=.284 | 0.176 (0.006) – 0.178 (0.005)  t(22)=2.54; *p*=.304 | 0.175 (0.004) – 0.188 (0.005)  t(18)=2.12; *p*=.006 | 0.177 (0.006) – 0.178 (0.003)  t(14)=2.43; *p*=.189 |
| AE = Aerobic Exercise; CCT = Computerized Cognitive Training; COMB = Combined Training; M = Mean ; SD = Standard Deviation | | | | |

1. **Nodes to networks mapping**

STable 1 tabulates the id, the full name of each brain area’s, its name according to AAL (Rolls et al., 2015), the located lobe, the abbreviated name and also the corresponding network used in our study. Here, we used the first 90 areas excluding the cerebellum brain areas (91-116).

The **Default Mode Network (DMN)** involves the following brain areas:

3 4 5 6 7 8 9 10 11 12 13 14 15 16 23 24 25

26 27 28 31 32 33 34 35 36 37 38 39 40 55 56 61 62

65 66 67 68 85 86 87 88 89 90

**The Occipital (O)** network involves the following brain areas:

43 44 45 46 47 48 49 50 51 52 53 54 55 56

The **Cingulo-Opercular (CO)** network involves the following brain areas:

29,30 31,32, 77,78

The **Sensory-Motor (SM)** network involves the following brain areas:

1 2 19 20 57 58

The **Fronto-Parietal (FP)** network involves the following brain areas:

13 14 15 16 17 18 35 36 59 60 61 62 63 64 65 66 67 68 69 70

**STable 1.** The anatomical regions defined in each hemisphere and their label in the automated anatomical labeling atlas - AAL (Rolls et al., 2015). Odd/even numbers refer to the left/right homologue brain areas.

**ID Region Description AAL Lobe Abbreviation**

1,2 Precentral gyrus Precentral Sensorimotor PreCG

3,4 Superior frontal gyrus,dorsolateral Frontal_Sup Frontal SFG

5,6 Superior frontal gyrus,orbital Frontal_Sup_Orb Frontal SForb

7,8 Middle frontal gyrus Front_Mid Frontal FMid

9,10 Middle frontal gyrus, orbital Front_Mid_Orb Frontal FMorb

11,12 Inferior frontal gyrus, opercular Front_Inf_Oper Frontal IFoper

13,14 Inferior frontal gyrus, triangular Front_Inf_Tri Frontal IFtri

15,16 Inferior frontal gyrus, orbital Front_Inf_Orb Frontal IForb

17,18 Rolandic operculum Rol_Oper Frontal Roloper

19,20 Supplementary motor area  Supp_motor_Area Sensorimotor SMA

21,22 Olfactory cortex Olfactory Frontal OLF

23,24 Superior frontal gyrus, medial Frontal_Sup_Med Frontal SFGmedial

25,26 Superior frontal gyrus, medial orbital Frontal_Med_Orb Frontal PFCventmed

27,28 Gyrus rectus  Rectus Frontal REC

29,30 Insula Insula Subcortical INS

31,32 Cingulate gyrus, anterior part Cingulate_Ant Frontal ACC

33,34 Cingulate gyrus, mid part Cingulate_Mid Frontal MCC

35,36 Cingulate gyurs, posterior part Cingulate_Post Parietal PCC

37,38 Hippocampus Hippocampus Temporal HIP

39,40 Parahippocampal Gyrus Parahippocampal Temporal PHG

41,42 Amygdala Amygdala Subcortical AMYG

43,44 Calcarine fissure and Calcarine Occipital V1

surrounding cortex (V1)

45,46 Cuneus Cuneus Occipital CUN

47,48 Lingual Lingual Occipital LING

49,50 Superior Occipital Gyrus Occipital_Sup Occipital SOG

51,52 Middle Occipital Gyrus Occipital_Mid Occipital MOG

53,54 Inferior Occipital Gyrus Occipital_Inf Occipital IOG

55,56 Fusiform Gyrus Fusiform Occipital FFG

57,58 Postcentral Gyrus Postcentral Sensorimotor PoCG

59,60 Superior parietal Gyrus Parietal_Sup Parietal SPG

61,62 Inferior parietal Gyrus Parietal_Inf Parietal IPG

63,64 Supramarginal Gyrus Supramarginal Parietal SMG

65,66 Angular Gyrus Angular Parietal ANG

67,68 Precuneus Precuneus Parietal PCUN

69,70 Paracentral Lobule Paracentral_Lobule Parietal PCL

71,72 Caudate nucleus Caudate Subcortical CAU

73,74 Lenticular nucleus,

Putamen Putamen Subcortical PUT

75,76 Lenticular nucleus,

Pallidum Pallidum Subcortical PAL

77,78 Thalamus Thalamus Subcortical THA

79,80 Heschl’s Gyrus Heschl Temporal HES

81,82 Superior Temporal Gyrus Temporal_Sup Temporal STG

83,84 Temporal Pole

Superior Temporal Gyrus Temporal_Pole_Sup Temporal TPOsup

85,86 Middle Temporal Gyrus Temporal_Mid Temporal MTG

87,88 Temporal Pole

Middle Temporal Gyrus Temporal_Pole_Mid Temporal TPOmid

89,90 Inferior Temporal Gyrus Temporal_Inf Temporal ITG

1. **Anatomical Locations of Brain Areas that constitute aDMN and pDMN.**

SFig.1 illustrates the anatomical locations and the relevant names of the ROIs that constitute the anterior and posterior DMN.


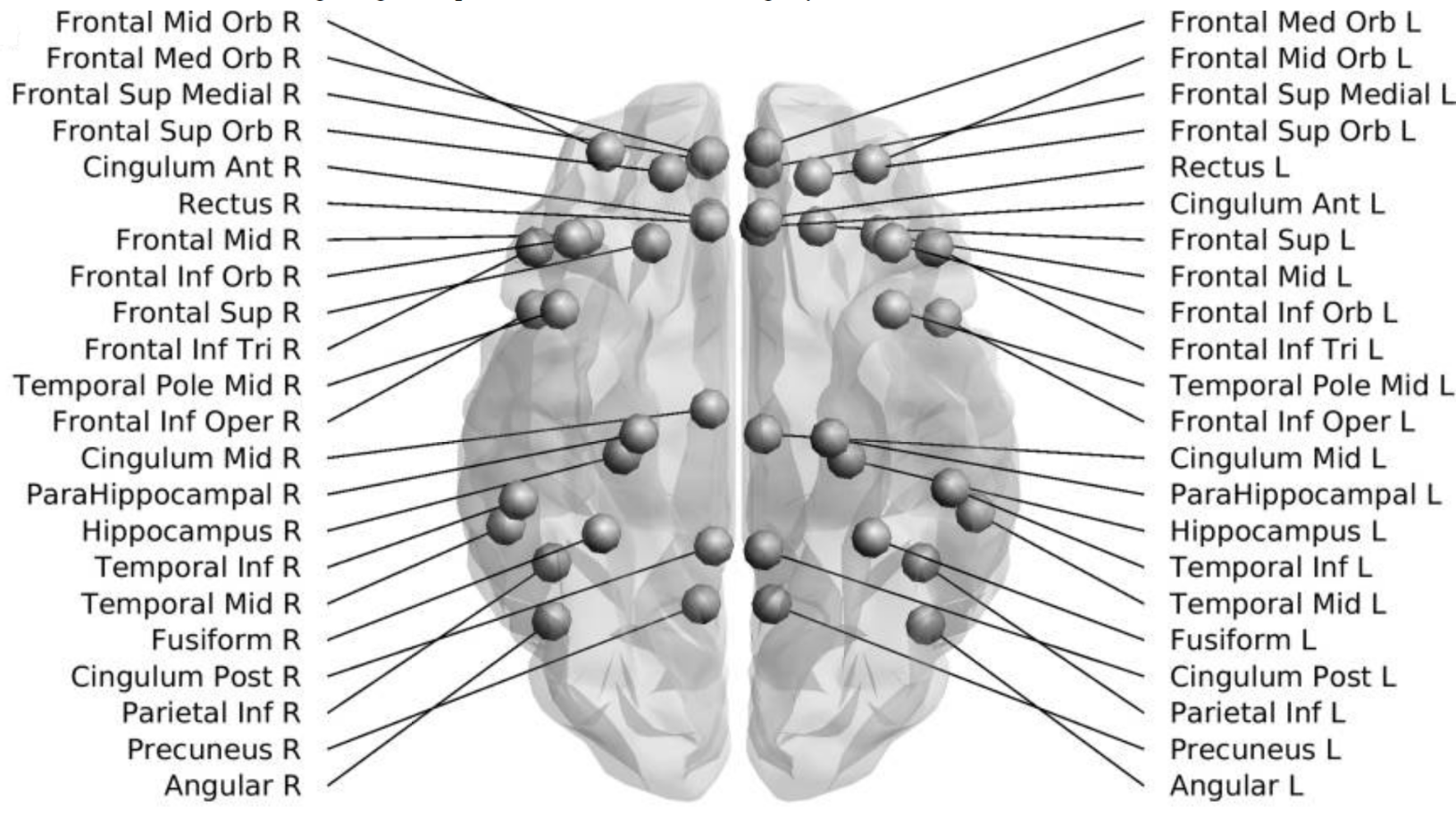


**SFig.1 .** Nodes included in the subnetwork analysis for the DMN and visual system. The top figure shows the 44 nodes included in the DMN adapted from the Power et al. 2011. The anterior part involves 12 x 2 hemispheres = 24 brain areas and the posterior part is constituted from 10 x 2 hemispheres = 20 brain areas.

1. **Group-averaged static functional connectivity networks (sFCN) at the baseline**

**SFig.2 illustrates** the group-averaged sFCN at the baseline for visual inspection.


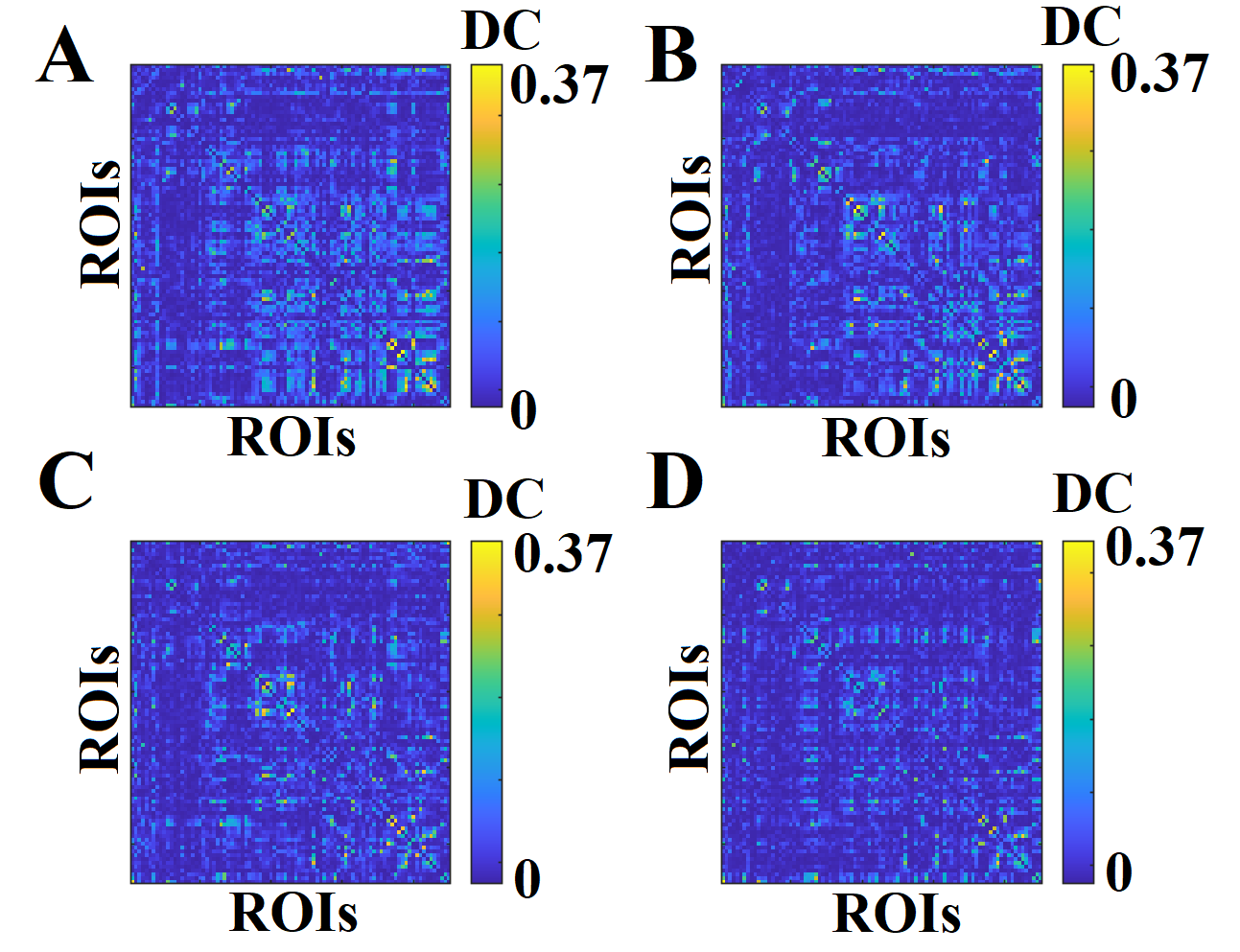


**SFig.2.** Group-averaged sFCN at the baseline normalized across the four groups for visual inspection and comparison.

1. HC
2. AE
3. CCT
4. COMB

**References**

1. Rolls, E. T., Joliot, M., and Tzourio-Mazoyer, N. (2015). Implementation of a new parcellation of the orbitofrontal cortex in the automated anatomical labeling atlas. Neuroimage 122, 1–5. doi: 10.1016/j.neuroimage.2015.07.075
2. Power JD, Cohen AL, Nelson SM, et al.. 2011. Functional network organization of the human brain. *Neuron* 72:665–678.
